# Effects of Remotely Supervised Home-Based High-Speed Bodyweight Resistance Training on Bradykinesia in Individuals With Parkinson Disease: Protocol for a Randomized Controlled Trial

**DOI:** 10.2196/84689

**Published:** 2026-05-04

**Authors:** Poliana do Amaral Yamaguchi Benfica, Aline Alvim Scianni, Carolina Luisa de Almeida Soares, Isabel Lopes Ribeiro, Pedro Vitor Casado, Christina Danielli Coelho de Morais Faria

**Affiliations:** 1 Department of Physical Therapy Universidade Federal de Minas Gerais Belo Horizonte Brazil

**Keywords:** Parkinson disease, bradykinesia, resistance training, telerehabilitation, clinical trial protocol

## Abstract

**Background:**

Exercises that involve increasing the speed of movements are beneficial for individuals with Parkinson disease (PD) and have the potential to reduce bradykinesia. High-speed bodyweight resistance training is accessible and versatile and can be performed anytime and anywhere, including at home. Furthermore, it is important to consider home exercises that enable treatment continuity and reduce barriers such as transportation difficulties and participation in physical exercise programs. However, we have not identified any studies that have conducted home-based high-speed bodyweight resistance training in individuals with PD.

**Objective:**

This protocol aims to investigate the effects of remotely supervised home-based high-speed bodyweight resistance training in reducing bradykinesia and improving mobility, muscle power, dynamic balance, and quality of life in individuals with PD.

**Methods:**

A randomized controlled trial will be performed with 1:1 allocation, blinded assessments, and an intention-to-treat analysis. Altogether, 46 individuals with PD, aged ≥50 years, who have bradykinesia and a sedentary or insufficiently active lifestyle, will be included. Participants will be randomly assigned to either (1) an experimental group (high-speed bodyweight resistance training) or (2) a control group (bodyweight intervention; usual speed). Both groups will perform a home-based, remotely supervised intervention, consisting of 60-minute individual sessions, 3 times per week over 12 weeks, with a trained physiotherapist. The primary outcome is bradykinesia of the lower limbs. The secondary outcomes are mobility, muscle power, dynamic balance, and quality of life. The effects of the training will be analyzed from the collected data by using the intention-to-treat analysis. Between-group differences will be measured by 2-way repeated measures ANOVA, considering the baseline, posttraining follow-up (primary time point), and 4-week follow-up (secondary time point).

**Results:**

Recruitment was conducted from October 2024 to October 2025 (n=46). Data collection is currently in the follow-up phase. The results are expected to be analyzed and submitted for publication by May 2026.

**Conclusions:**

The results of this trial will likely provide valuable new information on the effects of remotely supervised home-based high-speed bodyweight resistance training in reducing bradykinesia and improving mobility, muscle power, dynamic balance, and quality of life in individuals with PD. If confirmed, these findings may support the feasibility and effectiveness of an accessible home-based intervention delivered through telerehabilitation, potentially reducing barriers to rehabilitation.

**Trial Registration:**

ClinicalTrials.gov NCT06646523; https://clinicaltrials.gov/study/NCT06646523

**International Registered Report Identifier (IRRID):**

DERR1-10.2196/84689

## Introduction

Parkinson disease (PD) is a progressive neurodegenerative disorder that leads to increasing disability over time [1]. One of these disabilities is related to bradykinesia, which affects more than 80% of individuals with PD [2]. Bradykinesia refers to slowness in the execution of voluntary movements [3] with a decrement in amplitude or speed as movements continue (sequence effect) [4].

Bradykinesia affects the mobility of individuals with PD [5,6], contributing to difficulties in performing activities such as sitting, getting up from a chair, and walking [6]. Specifically, Parkinsonian gait is characterized by reduced stride length, decreased off-ground foot elevation, short stepping and shuffling, and reduced arm swinging [6]. Bradykinesia also impacts other outcomes such as muscle power [7], balance [8], and quality of life [5]. It is considered a problem in the speed with which individuals can activate muscles [8] and, therefore, can impact muscle power generation in individuals with PD. Moreover, bradykinesia is observable in reduced voluntary and reactive limits of stability [8], which can impair balance in this population.

Exercises that involve increasing the speed of movements are beneficial for individuals with PD and have the potential to reduce bradykinesia [9-11]. Studies using high-cadence cycling [9,10] and power training [11] have shown beneficial effects on bradykinesia in individuals with PD. However, the methodological quality of these studies has been classified as moderate [12], and there is not enough evidence currently available to recommend the implementation of these interventions in this population [12]. Therefore, it is necessary to conduct further studies with methodological rigor in the field, including investigating strategies that are considered feasible and accessible to individuals with PD. Both high-speed cycling [9,10] and muscle power training [11] use specialized equipment. This limits the access of individuals with PD to these treatment strategies, as these are costly pieces of equipment that are often not available in physiotherapy clinics.

A treatment strategy that involves increasing speed and has the potential to improve bradykinesia is high-speed bodyweight resistance training [13]. Bodyweight training refers to any exercise that involves using the individual’s own body mass as a means of resistance to perform work against gravity and ground reaction [13]. The main advantages of bodyweight training include its accessibility and versatility, as it can be performed at any time and place, including at home [13]. Studies that have investigated bodyweight resistance training in isolation in individuals with PD were not found. Considering other population groups, this training has previously been implemented in older individuals [14,15], individuals with obesity [16], and healthy individuals [17]. However, only 2 studies investigated the effects of high-speed bodyweight and found benefits on dynamic balance, muscle power, movement speed, and functional performance in older female individuals [14,15].

Finally, it is important to consider home-based exercises to increase accessibility and viability in individuals’ different social and economic contexts. Home-based exercises, in addition to enabling the continuity of treatment, reduce barriers such as transportation difficulties and participation in physical exercise programs [18]. Studies investigating home-based exercise in individuals with PD, mainly involving the modality via telerehabilitation, have already been shown to be safe and feasible [19-21].

Considering the importance of the speed component in individuals with PD affected by bradykinesia [2,22], and findings from previous studies involving this population [9-11], it is plausible that high-speed bodyweight resistance training may improve this outcome. Thus, the primary aim of this randomized controlled trial will be to investigate the effects of remotely supervised home-based high-speed bodyweight resistance training in reducing bradykinesia in individuals with PD. The secondary aim of this study will be to investigate the effects of remotely supervised home-based high-speed bodyweight resistance training in improving mobility, muscle power, dynamic balance, and quality of life in this population.

## Methods

### Design

**Figure 1:**
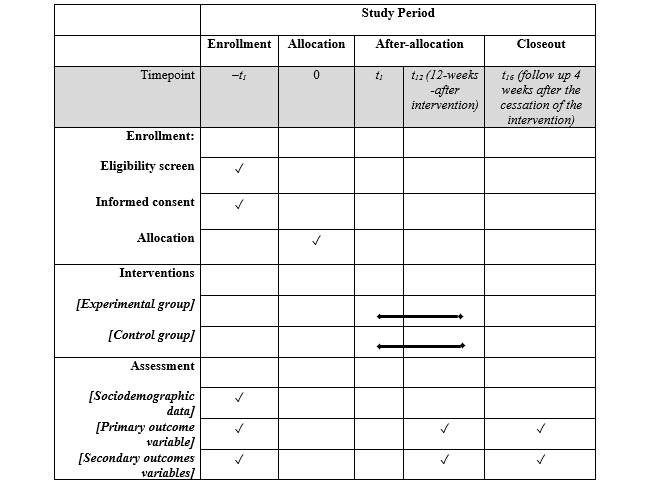
Schedule of enrolment, interventions and assessments.

**Figure 2:**
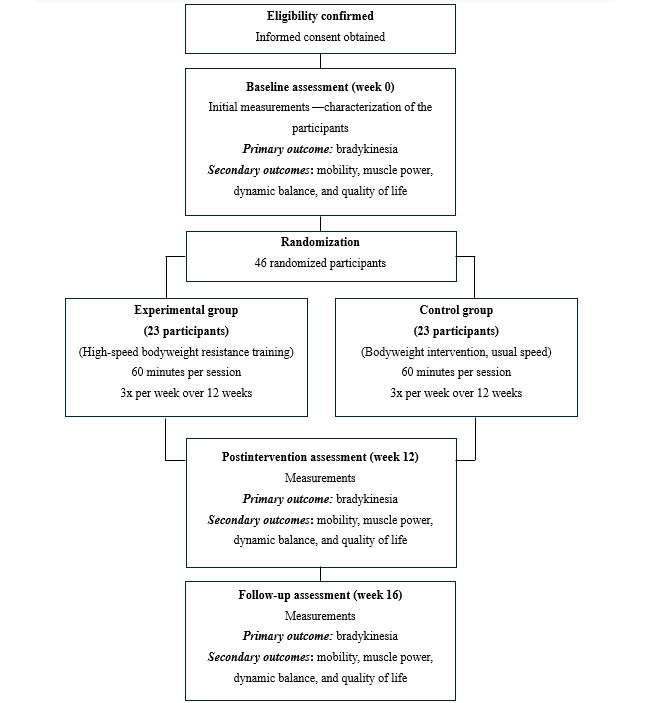
Flow diagram of the planned protocol.

A superiority parallel-group randomized controlled trial, 1:1 allocation, blinded assessments, and intention-to-treat analysis, will be performed in a community-based setting in the city of Belo Horizonte, Brazil. All participants will be informed of the study procedures and will provide written consent. Participants will be randomly assigned to the experimental or control group. Figure 1 shows the schedule of enrollment, interventions, and assessments [23]. Both groups will undertake intervention sessions of 60 minutes 3 times per week over 12 weeks [15]. Individual interventions will be performed at home and supervised remotely by a physiotherapist. Outcome measures will be collected at the Universidade Federal de Minas Gerais laboratory at baseline (week 0), after the intervention (week 12; primary time point), and at the 4-week follow-up (week 16; secondary time point) by the same examiner (Figure 2). This protocol has been developed in accordance with the SPIRIT (Standard Protocol Items: Recommendations for Interventional Trials) guidelines. This trial was registered at ClinicalTrials.gov (NCT06646523).

### Participants

Individuals will be recruited from the community through contact with health centers, research groups, university extension programs, and dissemination on social media. Individuals who express interest in participating will be contacted by a member of the research team and will be provided with detailed information about the study objectives, procedures, and eligibility criteria.

Eligibility will be determined through a 2-step screening process. An initial screening interview will be conducted by telephone to verify key inclusion and exclusion criteria. Individuals who meet the preliminary criteria will be invited to attend an in-person baseline assessment, during which clinical eligibility will be confirmed and written informed consent will be obtained prior to enrollment in the study.

Individuals will be included if they are 50 years or older, have idiopathic PD diagnosed by a neurologist, are classified between stages 1 to 3 of the modified Hoehn and Yahr Scale [24], present bradykinesia identified by items 3.8 and/or 3.14 of the motor examination of the Movement Disorder Society–sponsored revision of Unified Parkinson’s Disease Rating Scale (MDS-UPDRS) [25], are taking antiparkinsonian medication and are medically stable, are classified as inactive or insufficiently active [26], have the ability to walk independently without assistive devices, and have written medical permission to participate in the study. Bradykinesia will be established if participants score at least 1 point (out of 4) on item 3.8 (leg agility) and/or item 3.14 (global spontaneity of movement–body bradykinesia) of the MDS-UPDRS, which are validated measures of bradykinesia in PD [25]. Physical activity status will be determined using the Centers for Disease Control and Prevention classification: individuals reporting no exercise will be classified as inactive, whereas those performing insufficient exercise will be classified as insufficiently active [26].

Individuals will be excluded if they have cognitive impairments as determined by Mini-Mental State Examination cutoff scores (in points) adjusted for education level (illiterate: 13 points; elementary and middle school: 18 points; and high school: 26 points) [27]; have neurological, musculoskeletal, cardiovascular, or respiratory disorders that could affect their ability to perform the tests; have used deep brain stimulation; do not have access to the internet; and do not have a caregiver or family member who can assist during the intervention sessions.

### Participant Withdrawal

Participants may withdraw from the trial for any reason at any time. The principal examiner can withdraw participants from the study for safety purposes. A maximum of 10 consecutive days of nonperformance of the intervention will be allowed.

### Randomization

The randomization sequence will be computer-generated using a simple randomization method, in which each participant has an equal probability of being assigned to the experimental or control group. The sequence will be prepared prior to the start of the study by a trained research assistant who will not be involved in the study. The allocation will be maintained in sequentially numbered, sealed, opaque envelopes and stored in a locked cabinet and will not be accessible to investigators responsible for recruitment and assessment. After completion of the baseline assessment, the training therapist will open the next envelope in sequence to reveal the group allocation. Eligible participants will be randomly allocated to either the experimental or control group.

### Blinding

Due to the nature of the intervention, blinding of both participants and the training therapist to group allocation will not be feasible. However, measures will be implemented to minimize potential sources of bias. Standardized instructions and feedback will be provided to participants in both groups, and the frequency, duration, and amount of contact with the training therapist will be equivalent between groups. The training therapist will not be involved in outcome assessments. A trained examiner, blinded to group allocation, will collect all outcome measures, and the independent examiner responsible for statistical analyses will also remain blinded to group allocation. Participants will be instructed not to disclose their group allocation to the trained examiner.

### Interventions

Participants will be assigned to either the experimental group (high-speed bodyweight resistance training) or the control group (bodyweight intervention; usual speed). Before the start of the intervention, participants in both groups will receive a home visit to ensure that all procedures are properly explained and learned. During this visit, participants will be given the necessary equipment for the intervention: a digital blood pressure device for measuring blood pressure and heart rate before and after the intervention, and a step that will be used in one of the exercises. Furthermore, the duration of the exercises will be recorded. In the experimental group, exercises will be performed at a high speed, individualized according to each participant’s functional capacity, and progressing from their self-selected fast speed toward their maximum safe speed without compromising movement quality. In the control group, exercises will be performed at the participant’s usual self-selected pace, avoiding acceleration. Exercise speed will be continuously monitored by the principal examiner using a stopwatch, with immediate verbal feedback provided to ensure adherence. Cognitive strategies, including verbal feedback, auditory cues, and visual cues, will be applied as needed to reinforce the intended cadence and movement amplitude.

Participants in both groups will receive individualized home-based interventions via telerehabilitation, delivered through free text messaging and video platforms. Each session will consist of 5 minutes of warm-up (free active movements of the trunk and lower limbs), 50 minutes of exercises specifically targeting the lower limb muscles, and 5 minutes of cooldown (lower limb muscle stretching and relaxation breathing exercises) [14,15] and will be conducted in a one-to-one format under real-time supervision of the researcher responsible for delivering the intervention. Table 1 shows the proposed interventions for both groups.

**Table 1:** Exercises and protocol for both groups: experimental group (high-speed bodyweight resistance training) and control group (bodyweight intervention; usual speed).

Exercise^a^	Description	Protocol
Knee extension	Sitting position: extend the knee by lifting the leg forward	3 sets; 10 repetitions alternating the limbs; 30 s to 1 min of rest between sets of the same limb; maximum speeds for the experimental group and habitual speeds for the control group determined by the time of 1 set of 10 repetitions.
Knee flexion	Standing position: bend the knee to 90° by lifting the leg backward	3 sets; 10 repetitions alternating the limbs; 30 s to 1 min of rest between sets of the same limb; maximum speeds for the experimental group and habitual speeds for the control group determined by the time of one set of 10 repetitions; support from the UL^b^, if necessary.
Heel raise (plantar flexion)	Standing position: plantar flex the ankle by lifting the heel	3 sets; 10 repetitions alternating the limbs or bilaterally; 30 s to 1 min of rest between sets of the same limb; maximum speeds for the experimental group and habitual speeds for the control group determined by the time of one set of 10 repetitions; support from the UL, if necessary.
Hip extension	Standing position: extend the hip by lifting the thigh backward	3 sets; 10 repetitions alternating the limbs; 30 s to 1 min of rest between sets of the same limb; maximum speeds for the experimental group and habitual speeds for the control group determined by the time of one set of 10 repetitions; support from the UL, if necessary.
Hip flexion to 90°	Standing position: flex the hip and knee to 90° by lifting the thigh forward	3 sets; 10 repetitions alternating the limbs; 30 s to 1 min of rest between sets of the same limb; maximum speeds for the experimental group and habitual speeds for the control group determined by the time of one set of 10 repetitions; support from the UL, if necessary.
Hip abduction	Standing position: abduct the hip by lifting the leg laterally	3 sets; 10 repetitions alternating the limbs; 30 s to 1 min of rest between sets of the same limb; maximum speeds for the experimental group and habitual speeds for the control group determined by the time of one set of 10 repetitions; support from the UL, if necessary.
Trunk extension	Sitting position with the trunk leaning forward, extend the trunk backward	3 sets; 10 repetitions; 30 s to 1 min of rest between sets; maximum speeds for the experimental group and habitual speeds for the control group determined by the time of one set of 10 repetitions.
Stand up to sit	Sitting position: stand up and sit down in a chair	3 sets; 10 repetitions; 30 s to 1 min of rest between sets; maximum speeds for the experimental group and habitual speeds for the control group determined by the time of one set of 10 repetitions; support from the UL, if necessary.
Stair climbing	Standing position: lift the foot, step up onto a stair	3 sets; 10 repetitions alternating the limbs; 30 s to 1 min of rest between sets of the same limb; maximum speeds for the experimental group and habitual speeds for the control group determined by the time of one set of 10 repetitions; support from the UL, if necessary.
Squat	Standing position: flex the hips and knees bilaterally, lowering the body	3 sets; 10 repetitions; 30 s to 1 min of rest between sets; maximum speeds for the experimental group and habitual speeds for the control group determined by the time of one set of 10 repetitions; support from the UL, if necessary.
Walking in the home (5 m)	Standing position: walk 5 m on the level surface available in the home	3 sets; 4 repetitions; 30 s to 1 min of rest between sets; maximum speeds for the experimental group and habitual speeds for the control group determined by the time of one set of 4 repetitions.
Stand up and walk (3-m return)	Sitting position: stand up, walk 3 m, then return, and sit down	3 sets; 4 repetitions; 30 s to 1 min of rest between sets; maximum speeds for the experimental group and habitual speeds for the control group determined by the time of one set of 4 repetitions.

^a^In all exercises, the therapist will ensure the full range of motion.

^b^UL: upper limb.

The experimental intervention will be performed at maximum speed. During the initial home visit, the speed at which the participant can perform one set of each exercise at maximum speed will be timed. Speed-based progression will be determined every 4 weeks by increasing the number of repetitions in each set of exercises performed during subsequent home visits.

The control intervention will be performed at the usual speed. The same procedures that will be performed to determine the maximum speed in the experimental group will be used to determine the usual speed in the control group. There will be no progression in the speed of performing the exercises. However, participants in the control group will also receive a home visit from the principal examiner every 4 weeks to avoid bias related to the amount of attention given to participants in the experimental group.

### Procedures

A trained examiner, who will be blinded to group allocation, will collect the demographic, anthropometric, and clinical data, as well as the primary and secondary outcomes. All data will be collected in person at the *Laboratório de Estudos em Reabilitação Neurológica do Adulto—*NEUROLAB of the Universidade Federal de Minas Gerais.

### Primary Outcome Measure

Bradykinesia of the lower limbs will be measured using item 3.8 (leg agility), assessed bilaterally (right and left), and item 3.14 (global spontaneity of movement–body bradykinesia) of the motor examination of the MDS-UPDRS [25]. Each score ranges from 0 to 4, with 0 indicating normal function and 4 indicating severe impairment [25]. The primary bradykinesia outcome will be calculated as the sum of the bilateral scores for item 3.8 and the score for item 3.14, resulting in a composite score ranging from 0 to 12. Higher scores indicate more severe bradykinesia [28].

### Secondary Outcome Measures

Secondary outcomes will be mobility, muscle power, dynamic balance, and quality of life. Mobility will be assessed using the 10-meter walk test [29]. This test has adequate measurement properties and good clinical applicability for the assessment of individuals with PD [29]. Participants will be instructed to walk at both comfortable and maximal speeds in a 14-meter hallway, and the time taken to cover the central 10 meter will be timed [29,30]. A digital stopwatch will be used to measure 3 trials at comfortable and maximal speeds. The average of the 3 measurements will be used for analysis [29].

Muscle power will be assessed using the Five Time Sit to Stand (FTSTS) test [31]. This clinical test is easy and quick and presents adequate reliability to evaluate the functional performance of individuals with PD [32]. Participants will be instructed to perform FTSTS repetitions as rapidly as possible using a chair without armrests and with a standardized height [31]. The test will begin with the participant sitting on the chair with the upper limbs crossed over the chest and will finish when the participant sits on the chair after the fifth repetition [31]. The time needed to complete the task will be recorded with a stopwatch [31]. Only one repetition will be performed after 1 or 2 familiarization attempts with a rest time of 30 to 60 seconds [31]. FTSTS mean muscle power will be obtained by the product of FTSTS mean velocity and FTSTS mean force using the following equation:



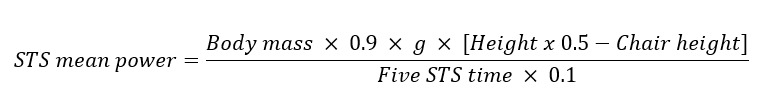



Dynamic balance will be assessed using the Mini-Balance Evaluation Systems Test (Mini-BESTest) [33]. The Mini-BESTest presents a version translated and cross-culturally adapted into Brazilian Portuguese, with adequate measurement properties (construct validity and test-retest reliability) for individuals with PD [34]. This test assesses changes in balance representing 4 domains of dynamic balance: anticipatory postural adjustments, postural responses, sensory orientation, and dynamic gait [33,34]. The Mini-BESTest is composed of 14 items, and for each item, the scores range from 0 to 2 [33,34]. A higher score indicates better performance [33,34].

Quality of life will be measured using the Parkinson’s Disease Questionnaire-39 (PDQ-39) [35]. The PDQ-39 was translated and adapted into Brazilian Portuguese and has adequate measurement properties for assessing individuals with PD [36]. The PDQ-39 is composed of 39 items divided into 8 dimensions: mobility, activities of daily living, emotional well-being, stigma, social support, cognition, communication, and bodily discomfort [35]. The score for each item ranges from 0 to 4, and for each dimension varies from 0 to 100 [35]. A higher score indicates a worse perception of quality of life by the individual [35].

### Data Monitoring and Management of Adverse Events

An independent researcher, blinded to group allocation, will be responsible for monitoring, recording, and reporting any adverse effects or discomfort, as well as for database management and statistical analyses. Participants will be instructed to immediately report any adverse events, which will be documented and classified according to severity and possible relation to the intervention. For safety, blood pressure, heart rate, and any adverse symptoms will be monitored during every session. Criteria for withholding or interrupting training have been established, such as excessively high or low blood pressure, discomfort or pain preventing performance, or ineffective medication. The principal examiner will supervise all exercise sessions, monitoring exercise intensity, participants’ safety, and adherence to the program. Decisions regarding whether an adverse event is related to the intervention will be made based on clinical judgment and temporal association with the exercise sessions. The trial will be temporarily suspended or terminated prematurely if serious adverse events occur that are deemed directly related to the intervention, or if safety concerns arise that cannot be mitigated. All participants will receive prior guidance to ensure a safe home environment before initiating the intervention. All adverse events will be reported to the institutional research ethics committee of the Universidade Federal de Minas Gerais in accordance with established guidelines.

### Sample Size

The sample size calculation was performed considering the primary outcome measure, bradykinesia (assessed using items 3.8, 3.9, 3.10, and 3.14 of the motor examination of the MDS-UPDRS) [25] based on data provided by a previous similar randomized controlled trial. The effect size for bradykinesia was derived from the study of Ni et al [11]. On the basis of a previous study [11] reporting a mean difference of –2.3 (SD 2.5), a sample size of 40 participants was required to achieve a power of 0.80 at a 2-tailed significance level (α=.05). Assuming a dropout rate of 15%, 46 participants will be recruited (23 per group).

### Statistical Analyses

All statistical analyses will be performed by an independent examiner blinded to group allocation using SPSS for Windows (IBM Corp). Baseline characteristics will be summarized by treatment group using descriptive statistics. Means (SD) or median (IQR) will be used for continuous variables according to analysis of normality (Shapiro-Wilk test). Frequency (percentage) will be used for categorical variables.

All analyses will follow the intention-to-treat principle, including all randomized participants in the groups to which they were originally allocated. The primary outcome will be the lower limb bradykinesia score, with the primary end point defined as the postintervention assessment at 12 weeks. Analytic approaches will be used to handle missing data, as recommended by Jakobsen et al [37]. The specific procedure will depend on the proportion of missing data, as stated by Jakobsen et al [37]. The statistical models will include a random intercept for participants and fixed effects for time (before intervention, after intervention, and follow-up), group (intervention and control), and group-by-time interaction. The primary analysis will focus on the group-by-time interaction effect. The level of significance will be set at 5%. Effect estimates will be reported considering the CIs (95% CI) for the between-group mean differences at each time point (week 12 minus week 0 and week 16 minus week 0). Secondary outcomes will be considered exploratory. Where applicable, adjustment for multiple comparisons will be performed using the Bonferroni method [38].

Adverse events will be summarized descriptively by treatment group, including the number and proportion of participants experiencing at least one event, as well as the frequency and severity of events.

### Ethical Considerations

This trial received approval from the institutional ethics review board (Comitê de Ética em Pesquisa, Universidade Federal de Minas Gerais, *Certificado de Apresentação para Apreciação Ética* —CAAE: 68350023.7.0000.5149, number 6.016.310, April 23, 2023). All participants will provide written informed consent.

All data collected during in-person assessments and remote supervision sessions, including exercise adherence records, blood pressure and heart rate logs, and reports of adverse events, will be recorded using standardized forms and entered into electronic databases. These data will be stored in a password-protected cloud-based platform linked to a dedicated project email account. Access to the database will be restricted to authorized members of the research team through individual log-in credentials and permission-controlled folders. Identifiable information will be stored separately from coded research data to ensure confidentiality. Data will be retained in accordance with institutional and national regulations.

After publication of the primary and secondary results, the anonymized dataset underlying the findings will be made available upon reasonable request to the corresponding author, in accordance with institutional ethics approval and applicable data protection regulations.

## Results

Recruitment began in October 2024 and concluded in October 2025. A total of 46 participants were enrolled in the study. Data collection is currently in the follow-up phase. Upon completion of follow-up assessments, data collection will be finalized. Data analysis has not yet been performed. We anticipate that the results will be compiled and submitted for publication by May 2026.

## Discussion

This trial is designed to investigate whether remotely supervised home-based high-speed bodyweight resistance training can reduce bradykinesia and improve other outcomes, including mobility, muscle power, dynamic balance, and quality of life in individuals with PD. We hypothesize that participants undergoing the intervention will demonstrate greater improvements in these outcomes compared with the control group. If confirmed, these findings may support the feasibility and effectiveness of an accessible home-based intervention delivered through telerehabilitation that does not require specialized equipment and could reduce barriers to rehabilitation, thereby increasing its clinical applicability [13,39,40].

Previous studies have demonstrated positive effects of speed-based interventions on bradykinesia in individuals with PD [9-11], but have not specifically investigated home-based high-speed bodyweight resistance training, which has shown beneficial effects in older adults [14,15]. Because the intervention in this study will be delivered through telerehabilitation, it may contribute to expanding evidence-based practice and, consequently, improve the care of individuals with PD.

The trial incorporates methodological strengths such as randomized allocation, blinded assessment, and intention-to-treat analysis, which enhance the internal validity of the results. By focusing on movement speed, a key aspect of bradykinesia, this study may inform the development of targeted exercise strategies to improve functional outcomes in PD.

However, some limitations must be acknowledged. Due to the nature of the intervention, neither participants nor therapists can be blinded to group allocation, and caregiver supervision will be required during training sessions to ensure participant safety. Moreover, the findings of this trial will be generalizable primarily to Brazilian individuals with PD who share similar characteristics with the study sample. Therefore, caution is needed in extending the results to broader populations.

If the intervention proves effective, future studies may explore long-term adherence and implementation in different health care settings and geographic regions. Overall, this protocol may provide valuable evidence on a feasible intervention to reduce bradykinesia in individuals with PD, contributing to the development of accessible rehabilitation strategies.

The findings of this trial are expected to be disseminated through peer-reviewed publications and presentations at national and international scientific conferences, contributing to the advancement of accessible rehabilitation strategies for PD.

## Appendix

Multimedia Appendix 1 SPIRIT (Standard Protocol Items: Recommendations for Interventional Trials) checklist.

## Abbreviations

FTSTS: Five Time Sit to Stand
MDS-UPDRS: Movement Disorder Society–sponsored revision of Unified Parkinson’s Disease Rating Scale
Mini-BESTest: Mini-Balance Evaluation Systems Test
PD: Parkinson disease
PDQ-39: Parkinson’s Disease Questionnaire-39
SPIRIT: Standard Protocol Items: Recommendations for Interventional Trials

## Data Availability

No datasets have yet been analyzed for the purposes of this protocol manuscript. Upon publication of the primary and secondary results, the anonymized dataset underlying the findings will be made available upon reasonable request to the corresponding author, in accordance with institutional ethics approval and applicable data protection regulations.

## References

[1] Bloem BR, Okun MS, Klein C (2021). Parkinson's disease. Lancet.

[2] Morris ME (2000). Movement disorders in people with Parkinson disease: a model for physical therapy. Phys Ther.

[3] Berardelli A, Rothwell JC, Thompson PD, Hallett M (2001). Pathophysiology of bradykinesia in Parkinson's disease. Brain.

[4] Bologna M, Paparella G, Fasano A, Hallett M, Berardelli A (2020). Evolving concepts on bradykinesia. Brain.

[5] Ellis T, Cavanaugh JT, Earhart GM, Ford MP, Foreman KB, Dibble LE (2011). Which measures of physical function and motor impairment best predict quality of life in Parkinson's disease?. Parkinsonism Relat Disord.

[6] Rodriguez-Oroz MC, Jahanshahi M, Krack P, Litvan I, Macias R, Bezard E, Obeso JA (2009). Initial clinical manifestations of Parkinson's disease: features and pathophysiological mechanisms. Lancet Neurol.

[7] Corcos DM, Chen CM, Quinn NP, McAuley J, Rothwell JC (1996). Strength in Parkinson's disease: relationship to rate of force generation and clinical status. Ann Neurol.

[8] Horak FB, Dimitrova D, Nutt JG (2005). Direction-specific postural instability in subjects with Parkinson's disease. Exp Neurol.

[9] Uygur M, Bellumori M, LeNoir K, Poole K, Pretzer-Aboff I, Knight CA (2015). Immediate effects of high-speed cycling intervals on bradykinesia in Parkinson's disease. Physiother Theory Pract.

[10] Ridgel AL, Ault DL (2019). High-cadence cycling promotes sustained improvement in bradykinesia, rigidity, and mobility in individuals with mild-moderate Parkinson's disease. Parkinsons Dis.

[11] Ni M, Signorile JF, Balachandran A, Potiaumpai M (2016). Power training induced change in bradykinesia and muscle power in Parkinson's disease. Parkinsonism Relat Disord.

[12] Cashin AG, McAuley JH (2020). Clinimetrics: Physiotherapy Evidence Database (PEDro) scale. J Physiother.

[13] Harrison JS (2010). Bodyweight training: a return to basics. Strength Cond J.

[14] Jaque-Gallardo C, Véliz-Campillay P, Cancino-López J (2019). Effect of a high-speed bodyweight resistance training on timed up and go and one leg stance in older women [Article in Spanish]. Rev Med Chil.

[15] Jaque C, Véliz P, Ramirez-Campillo R, Moran J, Gentil P, Cancino J (2021). High-speed bodyweight resistance training improves functional performance through maximal velocity in older females. J Aging Phys Act.

[16] Liu X, Gao Y, Lu J, Ma Q, Shi Y, Liu J, Xin S, Su H (2022). Effects of different resistance exercise forms on body composition and muscle strength in overweight and/or obese individuals: a systematic review and meta-analysis. Front Physiol.

[17] Kikuchi N, Nakazato K (2017). Low-load bench press and push-up induce similar muscle hypertrophy and strength gain. J Exerc Sci Fit.

[18] Allen NE, Sherrington C, Suriyarachchi GD, Paul SS, Song J, Canning CG (2012). Exercise and motor training in people with Parkinson's disease: a systematic review of participant characteristics, intervention delivery, retention rates, adherence, and adverse events in clinical trials. Parkinsons Dis.

[19] Flynn A, Preston E, Dennis S, Canning CG, Allen NE (2021). Home-based exercise monitored with telehealth is feasible and acceptable compared to centre-based exercise in Parkinson's disease: a randomised pilot study. Clin Rehabil.

[20] Vasconcellos LS, Silva RS, Pachêco TB, Nagem DA, Sousa CO, Ribeiro TS (2023). Telerehabilitation-based trunk exercise training for motor symptoms of individuals with Parkinson's disease: a randomized controlled clinical trial. J Telemed Telecare.

[21] Truijen S, Abdullahi A, Bijsterbosch D, van Zoest E, Conijn M, Wang Y, Struyf N, Saeys W (2022). Effect of home-based virtual reality training and telerehabilitation on balance in individuals with Parkinson disease, multiple sclerosis, and stroke: a systematic review and meta-analysis. Neurol Sci.

[22] Keus SH, Munneke M, Graziano M, Paltamaa J, Pelosin E, Domingos J, Brühlmann S, Ramaswamy B, Prins J, Struiksma C, Rochester L, Nieuwboer A, Bloem B (2014). European physiotherapy guideline for Parkinson's disease: developed with twenty European professional associations. KNGF/ParkinsonNet.

[23] Chan AW, Tetzlaff JM, Gøtzsche PC, Altman DG, Mann H, Berlin JA, Dickersin K, Hróbjartsson A, Schulz KF, Parulekar WR, Krleza-Jeric K, Laupacis A, Moher D (2013). SPIRIT 2013 explanation and elaboration: guidance for protocols of clinical trials. BMJ.

[24] Hoehn MM, Yahr MD (1967). Parkinsonism: onset, progression and mortality. Neurology.

[25] Goetz CG, Tilley BC, Shaftman SR, Stebbins GT, Fahn S, Martinez-Martin P, Poewe W, Sampaio C, Stern MB, Dodel R, Dubois B, Holloway R, Jankovic J, Kulisevsky J, Lang AE, Lees A, Leurgans S, LeWitt PA, Nyenhuis D, Olanow CW, Rascol O, Schrag A, Teresi JA, van Hilten JJ, LaPelle N (2008). Movement Disorder Society-sponsored revision of the Unified Parkinson's Disease Rating Scale (MDS-UPDRS): scale presentation and clinimetric testing results. Mov Disord.

[26] Centers for Disease Control and Prevention (CDC) (2001). Physical activity trends--United States, 1990-1998. MMWR Morb Mortal Wkly Rep.

[27] Bertolucci PH, Brucki SM, Campacci SR, Juliano Y (1994). The Mini-Mental State Examination in a general population: impact of educational status [Article in Portuguese]. Arq Neuropsiquiatr.

[28] Allen NE, Canning CG, Sherrington C, Fung VS (2009). Bradykinesia, muscle weakness and reduced muscle power in Parkinson's disease. Mov Disord.

[29] Lim LI, van Wegen EE, de Goede CJ, Jones D, Rochester L, Hetherington V, Nieuwboer A, Willems AM, Kwakkel G (2005). Measuring gait and gait-related activities in Parkinson's patients own home environment: a reliability, responsiveness and feasibility study. Parkinsonism Relat Disord.

[30] Tyson S, Connell L (2009). The psychometric properties and clinical utility of measures of walking and mobility in neurological conditions: a systematic review. Clin Rehabil.

[31] Alcazar J, Losa-Reyna J, Rodriguez-Lopez C, Alfaro-Acha A, Rodriguez-Mañas L, Ara I, García-García FJ, Alegre LM (2018). The sit-to-stand muscle power test: an easy, inexpensive and portable procedure to assess muscle power in older people. Exp Gerontol.

[32] Duncan RP, Leddy AL, Earhart GM (2011). Five times sit-to-stand test performance in Parkinson's disease. Arch Phys Med Rehabil.

[33] Franchignoni F, Horak F, Godi M, Nardone A, Giordano A (2010). Using psychometric techniques to improve the Balance Evaluation Systems Test: the mini-BESTest. J Rehabil Med.

[34] Maia AC, Rodrigues-de-Paula F, Magalhães LC, Teixeira RL (2013). Cross-cultural adaptation and analysis of the psychometric properties of the Balance Evaluation Systems Test and MiniBESTest in the elderly and individuals with Parkinson's disease: application of the Rasch model. Braz J Phys Ther.

[35] Jenkinson C, Fitzpatrick R, Peto V, Greenhall R, Hyman N (1997). The Parkinson's Disease Questionnaire (PDQ-39): development and validation of a Parkinson's disease summary index score. Age Ageing.

[36] Carod-Artal FJ, Martinez-Martin P, Vargas AP (2007). Independent validation of SCOPA-psychosocial and metric properties of the PDQ-39 Brazilian version. Mov Disord.

[37] Jakobsen JC, Gluud C, Wetterslev J, Winkel P (2017). When and how should multiple imputation be used for handling missing data in randomised clinical trials - a practical guide with flowcharts. BMC Med Res Methodol.

[38] Portney LG (2020). Foundations of Clinical Research: Applications to Evidence-Based Practice, 4th Edition.

[39] Langton B, King J (2018). Utilizing body weight training with your personal training clients. ACSMs Health Fit J.

[40] Rodríguez-Blázquez C, Forjaz MJ, Lizán L, Paz S, Martínez-Martín P (2015). Estimating the direct and indirect costs associated with Parkinson's disease. Expert Rev Pharmacoecon Outcomes Res.

